# Effects of Acidic Environments on Dental Structures after Bracket Debonding

**DOI:** 10.3390/ijms232415583

**Published:** 2022-12-09

**Authors:** Cristina Iosif, Stanca Cuc, Doina Prodan, Marioara Moldovan, Ioan Petean, Mîndra Eugenia Badea, Sorina Sava, Andrada Tonea, Radu Chifor

**Affiliations:** 1 Department of Prosthetic Dentistry and Dental Materials, “Iuliu Hatieganu” University of Medicine and Pharmacy, 32 Clinicilor Street, 400006 Cluj-Napoca, Romania; 2 Department of Polymer Composites, Institute of Chemistry “Raluca Ripan”, University Babes-Bolyai, 30 Fantanele Street, 400294 Cluj-Napoca, Romania; 3 Faculty of Chemistry and Chemical Engineering, University Babes-Bolyai, 11 Arany János Street, 400028 Cluj-Napoca, Romania; 4 Department of Preventive Dental Medicine, “Iuliu Hatieganu” University of Medicine and Pharmacy, Avram Iancu 31, 400083 Cluj-Napoca, Romania

**Keywords:** dental brackets, enamel surface, acid environment, shared bond test, surface roughness

## Abstract

Brackets are metallic dental devices that are very often associated with acidic soft drinks such as cola and energy drinks. Acid erosion may affect the bonding between brackets and the enamel surface. The purpose of this study was to investigate the characteristics of brackets’ adhesion, in the presence of two different commercially available drinks. Sixty human teeth were divided into six groups and bonded with either resin-modified glass ionomer (RMGIC) or resin composite (CR). A shared bond test (SBS) was evaluated by comparing two control groups with four other categories, in which teeth were immersed in either Coca-Cola^TM^ or Red Bull^TM^ energy drink. The debonding between the bracket and enamel was evaluated by SEM. The morphological aspect correlated with SBS results showed the best results for the samples exposed to artificial saliva. The best adhesion resistance to the acid erosion environment was observed in the group of teeth immersed in Red Bull^TM^ and with brackets bonded with RMGIC. The debonded structures were also exposed to Coca-Cola^TM^ and Red Bull^TM^ to assess, by atomic force microscopy investigation (AFM), the erosive effect on the enamel surface after debonding and after polishing restoration. The results showed a significant increase in surface roughness due to acid erosion. Polishing restoration of the enamel surface significantly reduced the surface roughness that resulted after debonding, and inhibited acid erosion. The roughness values obtained from polished samples after exposure to Coca-Cola^TM^ and Red Bull^TM^ were significantly lower in that case than for the debonded structures. Statistical results evaluating roughness showed that Red Bull^TM^ has a more erosive effect than Coca-Cola™. This result is supported by the large contact surface that resulted after debonding. In conclusion, the prolonged exposure of the brackets to acidic drinks affected the bonding strength due to erosion propagation into both the enamel–adhesive interface and the bonding layer. The best resistance to acid erosion was obtained by RMGIC.

## 1. Introduction

Various factors can affect the bonding of brackets during orthodontic treatment, such as enamel structure, bracket surface properties, poor operator technique, the patient’s behavior, and masticator forces [1]. The failure of bonding results in a longer period of time for treatment, which is why the cooperation of patients is necessary [2]. One essential factor is the patient’s oral hygiene, which can determine the accumulation of dental plaque, whether white spot lesions will occur, and whether the orthodontic therapy will be compromised [2].

The ideal cement used for bracket bonding should have good retention to the surface of the tooth, enough strength to resist and transmit the orthodontic forces during the course of therapy, and should be able to be removed without resulting in iatrogenic damage to the enamel [3]. A bonding strength of 5.9–7.8 MPa is recommended for orthodontic treatment [1].

Composite resins are frequently used for bonding brackets due to their good aesthetic and bond strength [4]. However, to bond with composite resins we need a completely dry surface, and the patient’s compliance is essential [5]. Due to the fixed appliance, and depending on the behavior of the patient regarding oral hygiene, the prevalence of plaque accumulation tends to increase during orthodontic treatment [6]. This is why the risk of decalcification is larger during the therapy and tends to occur around the bracket, in particular in the gingival area, which is more difficult to clean [7].

Demineralization can occur as early as one month after the beginning of treatment [6] and can result in white spot lesions or enamel cavities in severe cases. Enamel demineralization after orthodontic treatment can appear in up to 50% of patients [2]. It has been demonstrated that there is a possibility for the initial damage to be repaired with the use of fluoride, which has the capacity to re-mineralize teeth, resulting in an anti-caries effect [7].

In other words, when the brackets are bonded with cement resins it can result in white spot lesions and the risk of enamel damage after bracket removal, which can reduce the success of the treatment [4]. As a result, the use of other materials, such as glass ionomers, has been suggested. The advantages of glass ionomers are that they can be used for bonding in cases with moisture contamination, such as when we cannot realize the proper isolation of the teeth [2], and the fact that they are fluoride-releasing, with the potential to decrease the demineralization of the surface of the enamel around the bracket [6].

However, glass ionomers have a lower bond strength (2.37–5.5 MPa), which will frequently result in bond failure [8], and therefore limits their clinic use.

Hybrid glass ionomer cements were developed to combine the adequate bond strength of cement resins with the fluoride-releasing properties of glass ionomers [6]. Ghubaryi, et al. [9] demonstrated that the bond strength is initially lower, but will increase after 24 h to 5.39–18.9 MPa; such hybrid cements have been successfully used in orthodontic therapy.

Enamel demineralization is a severe complication of orthodontic treatment and is the result of a decrease in salivary pH [5]. If its pH is lower than 5, saliva is sub-saturated in fluoroapatite and hydroxyapatite, and this creates a medium for demineralization. Behavioral factors, such as the pH of drinks consumed, and buffering of saliva can result in enamel demineralization and dental erosion [5];in such cases, the bond between bracket and enamel will be affected and the success of the treatment will be compromised.

More people, especially children, and teenagers, are frequently consuming soft drinks [10]. This kind of beverage includes all drinks except alcohol, mineral water, fruit juices, tea, coffee, or milk-based drinks, and may or may not be carbonated [5]. The carbonated beverages are more aggressive, because of their carbonic acid content [11].

Soft drinks have a negative impact on the structure of enamel because of their content of sugar and citric acid, which are very erosive and will degrade the surface of the tooth around the brackets [1]. The consumption of soft drinks results in a pH lower than 5.5, which can then lead to dental erosion. Dental erosion is a defect on the surface of enamel resulting from exposure to acids and will compromise the bond between the bracket and the tooth, because healthy enamel is essential for the retention of the bracket.

Coca-Cola is the most popular soft drink, and one of the three best-known beverages [12]. Red Bull is the most frequently consumed energy drink. One of the best-known slogans in the United States is “Red Bull gives you wings”, because of the effects of the drink on mental and athletic performance due to its caffeine content. They also might influence the dental material color [13] and behavior due to the acid action at the body temperature [14]. The dental plaque proliferation on the bonding layer also might be influenced by acid components of mentioned soft drinks [15].

The purpose of this study is the determination of these two soft drinks’ effects on the shear bond strength and microstructural aspects regarding orthodontic metal brackets bonded with two different materials: cement resin (Transbond Color Change) and RMGIC (Fuji Ortho LC).

## 2. Results

### 2.1. Shear Bond Strength

The adhesion was evaluated by recording the shear strength according to the standard procedures [16,17], which is generally used to evaluate adhesive systems in the laboratory. Generally, the comparison between materials or bracket application methods is justified, but the extrapolation of these results in the clinical situation is not easy. The results obtained by us for CR and RMGIC are presented in Table 1. The highest value for SBS was recorded for the RMGIC sample exposed to Saliva (7.65 MPa), followed by the CR control sample, exposed only to artificial saliva (5.20 MPa), and the lowest value for bond strength was recorded for the CR sample exposed to Cola (2.70 MPa). The other sample groups all registered SBS of approximately 3 MPa.

Statistical Analysis: The results of the bracket adhesion test for all six investigated teeth groups showed different statistical significance, with a *p*-value = 2.77933 × 10^−4^. Following the Tukey test, it was evident that different statistical significances are only between RMGIC immersed in artificial saliva and the other groups tested (RMGIC in COLA, CR in COLA, CR in HELL, CR in artificial saliva).

The Anova test obtained a value of *p* = 0.00906, regarding the breaking strength of the bracket with little different statistics between groups: RMGIC immersed in artificial saliva and CR in COLA; RMGIC in artificial saliva and RMGIC in COLA. The same differences between groups are also obtained in the case of the maximum strength accepted, with a *p* = 0.01026.

### 2.2. Scanning Electron Microscopy SEM

**Figure 1:**
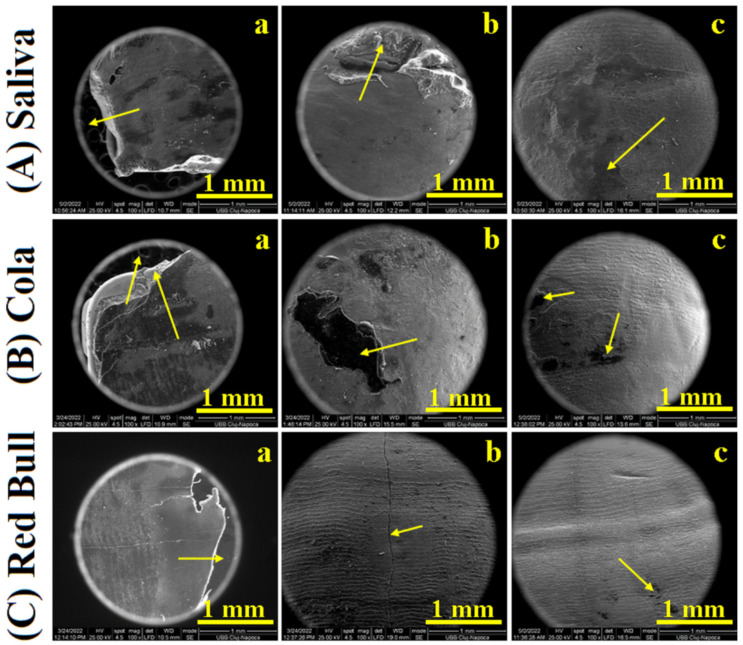
SEM images of CR samples: (**a**) bracket after debonding, (**b**) enamel after debonding, and (**c**) debonded enamel after polishing; stored in Saliva (**A**), exposed to Cola (**B**) and exposed to Red Bull^TM^ (**C**).

**Figure 2:**
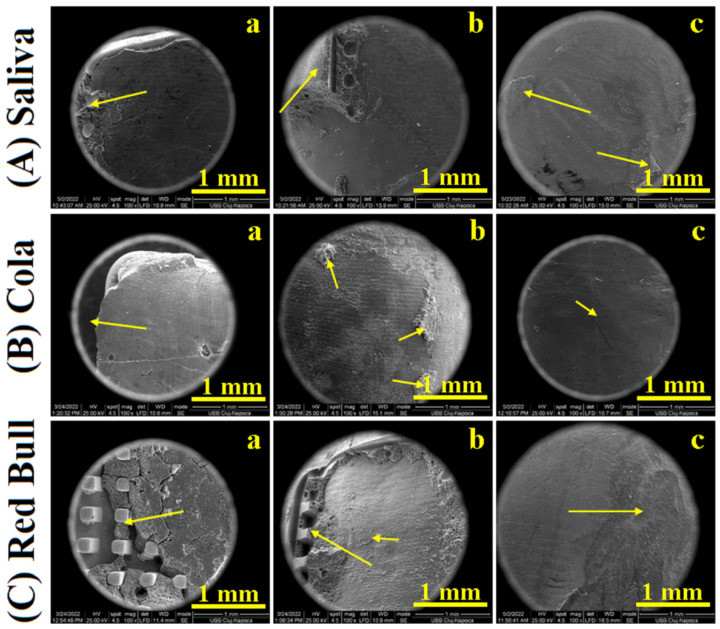
SEM images of RMGIC samples: (**a**) bracket after debonding, (**b**) enamel after debonding, and (**c**) debonded enamel after polishing; stored in Saliva (**A**), exposed to Cola (**B**) and exposed to Red Bull^TM^ (**C**).

SEM images were taken on low magnification to observe all morphological details regarding the shearing of the adhesion layer between brackets and enamel. Its failure under shearing stress generated important morphological details that are necessary to explain the variation related to the data in Table 1. The images resulting from CR samples are presented in Figure 1 and the ones for RMGIC samples are presented in Figure 2.

CR control sample exposed to the artificial saliva reveals a complex morphology adhesive layer shearing. The bracket base, Figure 1(Aa), is almost covered with an adhesive having a relatively uniform surface (from a macroscopic point of view). It covers about 60% of the bracket base. The shearing stress during failure caused adhesive layer fragmentation with complete detachment from the other 40% of the bracket base which is clearly observed in the direction of the yellow arrow in Figure 1(Aa). On the opposite, the enamel surface after debonding is macroscopically smooth for about 60%, and the other 40% presents adhesive fragments as indicated with the arrow in Figure 1(Ab). The polishing effect removes the adhesive debris and small unevenness which conducts to a plain surface of good macroscopic quality, Figure 1(Ac). There appear some small microscopic irregularities and scratches which need a detailed microscopic investigation using the atomic force microscope (AFM).

Figure 1(Ba) presents the bracket base of the CR sample exposed to Coca-Cola^TM^. Adhesive failure after debonding leads to a complex microstructure formed by: a large part of the surface covered with an adhesive of about 70% being relatively uniform and 30% of the surface being irregular due to the adhesive fragmentation. It occurs in two steps: a thinner rest of adhesive is observed on about 15% of the surface due to its internal failure under debonding and about 15% of the free bracket base, see the arrow in Figure 1(Bb). The polishing treatment removes the adhesive debris from the enamel surface in a good manner but some residual adhesive traces are still present, Figure 1(Bc).

Red Bull^TM^ exposure of the CR sample certainly influences the bracket debonding features, Figure 1C. The bracket failure occurred at the enamel-adhesive interface, most of the adhesive being attached to the bracket base on 85% of the surface, the rest being fragmented and completely detached from the debonded parts; see the arrow in Figure 1(Ca). The enamel surface is completely free of adhesive at the macroscopical view, and some microscopic traces may occur. A significant crack in the enamel is observed and indicated with the arrow in Figure 1(Cb). The polishing procedure conducts to a smoother surface free of cracks and only very small traces of adhesive areobserved, Figure 1(Cc).

The morphology of the RMGIC sample kept in the artificial saliva after debonding is presented in Figure 2A. Bracket failure during debonding occurred at the enamel adhesive interface. Therefore, the base of the bracket is almost completely covered by the adhesive of about 88% of the surface only 12% of the bracket base being visible on the side indicated with the yellow arrow, Figure 2(Aa). The enamel surface after debonding, Figure 2(Aa), reveals a macroscopic uniform surface which is covered with adhesive only on about 12% of its surface as indicated with an arrow in Figure 2(Ab). Some footprints from the bracket base are visible, imprinted in the adhesive on the observed area. The polishing procedure is required to remove this debonding debris on the enamel surface. It was an improvement during the restoration attempt as observed in Figure 2(Ac). Some irregularities on the enamel surface are observed, due to the adhesive that was not polished enough.

Coca-Cola^TM^ exposure to the RMGIC sample influences the debonding parts morphological aspect as observed in Figure 2B. The adhesive failure occurs mainly on the enamel interface assuring proper preservation of the parts. Most of the adhesive layer remains attached to the bracket, Figure 2(Ba), covering about 83% of its base. The other 17% corresponds to the free view of the bracket base as shown with the yellow arrow. The adhesive in this area was broken during debonding and fell away. The enamel surface, Figure 2(Bb), presents a uniform surface after debonding, only small areas with adhesive traces were observed, Figure 2(Bb). The debonding structures and erosion marks were very well removed by the polishing treatment, only few scratches being visible in Figure 2(Bc).

RMGIC samples exposed to Red Bull^TM^ after debonding presents a very interesting morphology. Almost 50% of the bracket base surface is covered with an adhesive having internal fissures and the other part is visible, Figure 2(Ca). This internal fragmentation of also influences the morphology of the enamel surface, Figure 2(Cb). There are significant adhesive deposits on 15% of the surface and several small traces as indicated with arrows. A polishing procedure is absolutely necessary. Figure 2(Cc) evidenced on the left side of the observation field a very well-restored area of the enamel (about 55% of the surface) and on the right side appears several remaining defects such as traces of adhesive and erosion features.

### 2.3. Atomic Force Microscopy AFM

**Figure 3:**
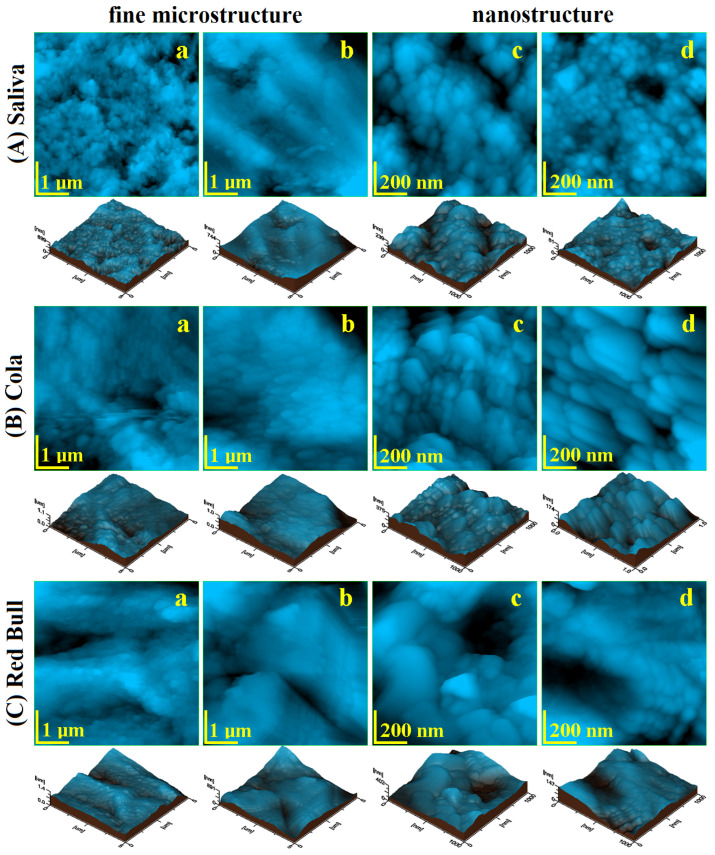
AFM imaging of enamel after CR debonding: fine microstructure before polishing (**a**) and after polishing (**b**); nanostructure before polishing (**c**) and after polishing (**d**); stored in Saliva (**A**), exposed to Cola (**B**), and exposed to Red Bull^TM^ (**C**).

**Figure 4:**
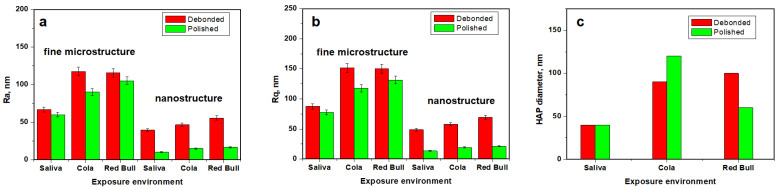
The evolution of the parameters measured with AFM for CR samples: (**a**) Ra, (**b**) Rq, and (**c**) nano HAP diameter.

The fine microstructure of debonded CR sample exposed to saliva is presented in Figure 3(Aa). The surface topography evidenced several HAP nanostructural units pulled out during the debonding process forming some depressions with irregular margins and the diameter ranging from 300 to 1000 nm. There are also some adhesive debris traces situated on the highest zones of the observation field. The Coca-Cola^TM^ exposure causes a pronounced decaying of the enamel fine microstructure due to the progressive enlarging of the depressions and significant weight loss associated with HAP nanoparticles loss, Figure 3(Ba). It causes a significant increase in surface roughness, Figure 4a,b. The exposure to Red Bull^TM^ energy booster juice leads to an advanced decaying of the enamel surface by pronounced enlarging of the depressions, their aspects becoming similar to the desert dunes, Figure 3(Ca). These fine microstructural aspects are in good agreement with SEM observation in Figure 1(Ab,Ac). The surface roughness value increases significantly because of the observed decayed features.

The polishing treatment leads to a proper CR enamel surface smoothness which is considerably improved, Figure 3(Ab). The adhesive debris traces from the enamel surface were completely removed by polishing and the topographic depressions (caused by HAP units pulling out during debonding) are considerably attenuated. Therefore, the roughness value significantly decreases in Figure 4.

The enamel surface compactness resulting after the polishing treatment determines a better resistance to acid erosion due to the removal of the depressions which act as decaying promoters. Thus, Coca-Cola and Red Bull^TM^ found a compact surface without faults to be penetrated and enlarged. It means that the erosive elements within the mentioned juices must generate the erosion faults prior to being enlarged. Therefore, the roughness values after exposure are lower than the ones observed for the unpolished samples, Figure 4. The Coca-Cola^TM^ exposure generates a surface topography having a washout aspect but with low penetration in-depth, Figure 3(Bb). The surface topography is more rugged after Red Bull^TM^ exposure as observed in Figure 3(Cb). Thus, the second aspect of the null hypothesis is rejected because the roughness resulting after Red Bull is significantly greater than the one caused by Cola, Figure 4.

The nanostructure of the CR enamel sample resulting after debonding is presented in Figure 3(Ac). The observed topography is quite rugged because of the depressions formed by HAP nanostructural units pulling out during debonding. In addition tothese certainly affected areas, there are remarked compact portions of healthy enamel featuring nanostructural units of about 40 nm diameter well bonded one to another by the protein binder. The exposure to Coca-Cola^TM^ causes a widening of the above-mentioned depressions along with an advanced erosion of the areas initially unaffected, Figure 3(Bb). Therefore, the diameter of the HAP nano-structural units increases in the range of 60–90 nm, Figure 4c. The exposure to Red Bull^TM^ leads to a more advanced decaying of the nanostructure, Figure 1(Cc). We remark on the increase in the depression’s depth along with advanced demineralization signs such as washout surfaces and increased diameter of the nanostructural units around 100 nm. The roughness values are significantly increased as observed in Figure 4a,b.

The nanostructure resulting from the CR enamel after the polishing procedure presents quite a uniform topography, Figure 3(Ad), which corresponds to a compact surface with HAP nanostructural units very well attached one to another. Their diameter is situated at about 40 nm in good agreement with the values for the healthy enamel [18,19]. The surface roughness significantly decreases as observed in Figure 4a,b, also being in good agreement with the data in the literature for healthy enamel [18,19].

Coca-Cola^TM^ exposure causes an advanced erosion of the HAP nanostructural units from the CR enamel surface but without generating the decaying depressions, Figure 3(Bd). The fact determines the severe increase in the nanostructural unit diameter from 40 nm to over 120 nm. The exposure to Red Bull on the CR enamel surface leads to more advanced decay as observed in Figure 3(Cd).We observe an undulated topography associated with HAP crystallites with deep erosion marks and traces of weight loss. This fact leads to increased values of roughness compared to the Cola exposure. Therefore, the null hypothesis was rejected as a nanostructural point of view.

**Figure 5:**
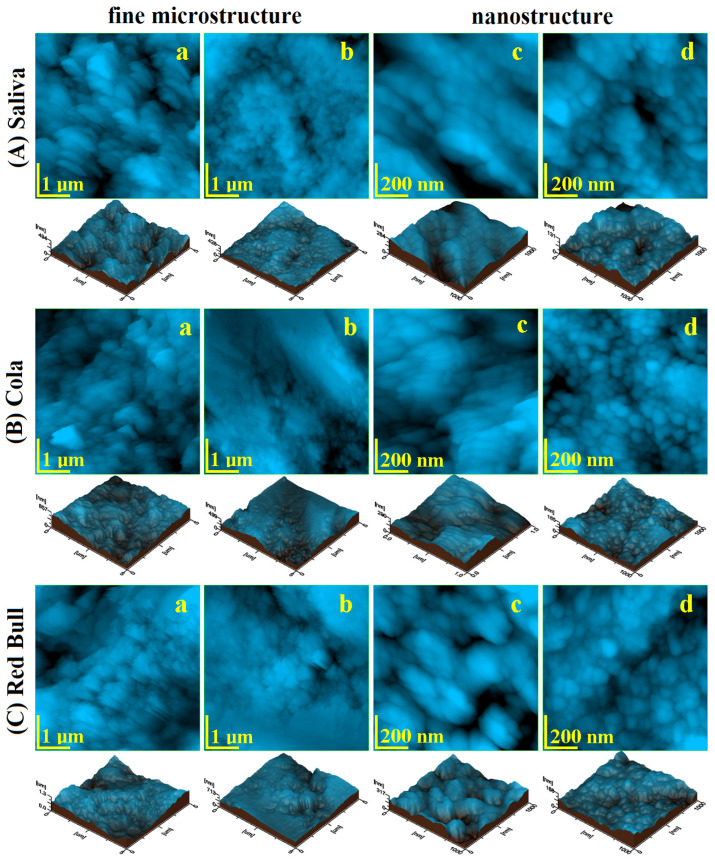
AFM imaging of enamel after RMGIC debonding: fine microstructure before polishing (**a**) and after polishing (**b**); nanostructure before polishing (**c**) and after polishing (**d**); stored in Saliva (**A**), exposed to Cola (**B**), and exposed to Red Bull^TM^ (**C**).

**Figure 6:**
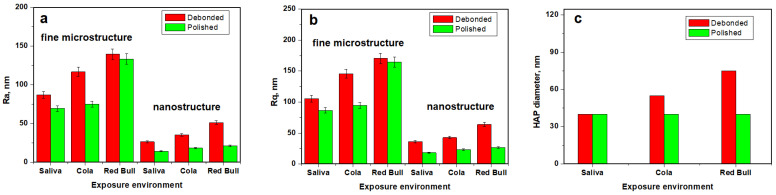
The evolution of surface parameters measured with AFM for RMGIC samples: (**a**) Ra,(**b**) Rq, and (**c**) nano HAP diameter.

Bracket debonding from RMGIC adhesion on the enamel stored in the artificial saliva leads to a very rugged surface. It presents a mixed characteristic resulting from the topographic combination of the submicron adhesive traces and depressions formed by HAP nanostructural units pulling out during debonding, Figure 5(Aa). These topographic features have the nature to generate a relatively high surface roughness as observed in Figure 6a,b.

Advanced acid erosion on the RMGIC debonded sample exposed to Coca-Cola^TM^, is remarked in Figure 5(Ba). The adhesive traces present a better resistance to the erosive effect of the phosphoric acid, meanwhile, HAP nanostructural units are deeply eroded with evidence of significant weight loss. The roughness values proved to be greater than the ones observed for the control sample. Red Bull^TM^ proves to be more aggressive than Cola by the significant deepening of the acid erosion depressions, Figure 5(Ca). Such an erosive effect leads to the greater roughness values reached in the present research, Figure 6a,b.

RMGIC enamel sample polishing is an absolutely necessary step for the adhesive submicron traces removal as well as for the depressions (formed by HAP nanostructures unit pulling out during debonding) attenuation. The topography of the sample stored in artificial saliva after polishing treatment is smoother and more compact as observed in Figure 5(Ab). The complete removal of submicron adhesive traces is observed along with a very compact disposal of the Hap nanostructural units similar to the typical structures in the healthy enamel according to the data in the literature [19,20]. The mentioned depressions are well attenuated and their marks are almost unobservable. A direct consequence of these facts is the significant decrease in roughness, as observed in Figure 6a,b.

The exposure to Coca-Cola^TM^ of RMGIC polished enamel presents a significant tendency of the acid etching of the HAP pulling out depressions to form acid erosion depressions, Figure 1(Bb). It is a matter of the topography disturbance that increases the roughness value. The exposure to Red Bull^TM^ causes slightly enhanced erosion features than those observed for Cola, Figure 1(Cb) and to the formation of several deep valleys such as the one observed in the upper right side of the topographical image in Figure 1(Cb). These additional features lead to an additional increase in roughness, Figure 6.

Considering the AFM evidence, we remark that the polishing treatment on the RMGIC enamel samples is beneficial to prevent the propagation of the acid erosion features into the profoundness of the samples and consequently to prevent the excessive increase in roughness. The resistance of RMGIC adhesive traces to the acid erosion especially to the Red Bull is observed.

The RMGIC enamel sample nanostructure after debonding and storage in artificial saliva presents some small areas of pulled-out nano HAP units but also some submicron adhesive traces which remain from the adhesion interface, Figure 1(Ac). Therefore, the HAP nanostructural unit diameter is about 40 nm, Figure 6c. The exposure of this surface to Coca-Cola^TM^ leads to the enlarging of the depressions by the acid erosion of exposed enamel and due to the tendency to penetrate depth. The nano HAP units present on the surface are significantly affected by the acid causing their diameter to increase to about 55 nm as observed in Figure 5(Bc). Thus, significant roughness increasing occurs. The exposure to Red Bull^TM^ generates a more affected nanostructure by the of rugged topography with an aspect of eroded sand dunes, Figure 5(Cc). The nano HAP unit diameter is situated in the range of 60–90 nm; it indicates that they are close to their material disintegration which is able to cause weight loss. It has a strong influence on the roughness increasing. Therefore, the null hypothesis is totally rejected by the RMGIC enamel samples nanostructure after debonding.

The RMGIC polished enamel stored in artificial saliva nanostructure is considerably improved, Figure 5(Ad). The surface topography is more uniform than before treatment due to the adhesive trace removal and attenuation of the nano HAP pulling out depressions. The enamel morphology revealed a nano Hap unit with a diameter of about 40 nm, in good agreement with the data in the literature for healthy enamel [20,21], which are well welded one to another. In fact, this sample is so good that presets the lowest roughness among all samples in the current study, Figure 6a,b. The Coca-Cola^TM^ and Red Bull^TM^ exposure of the polished RMGIC samples leads to very interesting topographies, Figure 5(Bd,Cd). The microstructure general aspect is well preserved with a nano HAP unit of about 40 nm and compactly organized. Only a small erosion of their boundaries is observed which is significantly pronounced for Red Bull compared to Cola. A small roughness increase was observed. In consequence, the null hypothesis is also rejected completely for the RMGIC enamel polished samples.

Roughness statistical analysis for the investigated samples is centralized in Table 2 and Table 3.

The AFM statistical results of the enamel fine microstructure after debonding, but before polishing, present no significant statistical differences after the exposure to Cola or Red Bull in both cases, when the brackets were bonded with composite resin or glass ionomer modified with resins. For the enamel fine nanostructure, statistical differences do not exist between the values after exposure to Cola or Red Bull^TM^ before polishing when was used composite resin or glass ionomer modified for bonding.

In this case as well, the AFM statistical results of the enamel fine microstructure and nanostructure after debonding and polishing present no significant statistical differences after exposure to Cola or Red Bull^TM^, for composite resin and glass ionomer modified.

There are no significant statistical differences when comparing the enamel fine microstructure both before and after polishing for Ra and Rq in all three sample groups (the control samples immersed in artificial saliva, the samples exposed to Cola, and those exposed to Red Bull^TM^), as all *p* values are higher than 0.05. Ra for: control sample—*p* = 0.33, Cola exposure—*p* = 0.40; Red Bull^TM^ exposure *p* = 0.78; Rq for control sample—*p* = 0.24, and Cola exposure—*p* = 0.40; Red Bull^TM^ exposure *p* = 0.68.

There are significant statistical differences when comparing the enamel nanostructure both before and after polishing for UM Ra and Rq in all three sample groups (the control samples immersed in artificial saliva, the samples exposed to Cola, and those exposed to Red Bull^TM^): Ra for control sample—*p* = 0.04, Cola exposure—*p* = 9.02897 × 10^−4^; Red Bull^TM^ exposure—*p* = 7.23641 × 10^−4^; Rq for control sample—*p* = 0.04, Cola exposure—*p* = 5.9025 × 10^−4^; Red Bull^TM^ exposure *p* = 0.00156.

## 3. Discussion

Oral hygiene, orthodontic bonding technique, and nutrition are the factors determining the development of dental erosion during orthodontic treatment [11,22,23]. It is a matter of complex interaction between the enamel surface and bracket towards the adhesive layer. Poor oral hygiene causes food residue deposits around the brackets and bonds which generate erosive compounds during the putrefaction process. Poor bonding technique will generate weaker adhesion of the bracket to the enamel and may generate local erosion [24]. Nutrition is very often associated with acidic food and drink ingestion. The acid compounds within the masticated and ingested aliments will decrease the retention of the brackets and will affect the results of the therapy.

The percentage of teenagers who are consuming soft drinks has increased in the last few years. This is the reason we chose to use Coca-Cola^TM^, the most famous of carbonated drinks, and Red Bull^TM^, the most well-known energy booster drink. Red Bull^TM^ contains citric acid and Coca-Cola^TM^ phosphoric acid, both of which are used in acid etching for bonding orthodontic brackets [10]. These acids are also reported in the literature to be the main cause of enamel surface demineralization [25,26]. It is demonstrated that even short periods of consuming soft drinks diminish the enamel microhardness and increase the roughness. It is crucial to limit exposure to this type of beverage to prevent erosive lesions [1,26]. Acidic drinks reduce oral cavity pH lower than 5.5 which will favors the dissolution of hydroxyapatite and fluor-apatite within the teeth enamel [5] and limits the re-mineralizing effect of the saliva. The tooth enamel is a non-remodeling tissue, for this reason, any changes that occur on its surface will be permanent [27].

The aim of this study is to observe how much shear bond strength can be affected by the consumption of soft drinks and for what type of material (composite resin or glass ionomer modified) it can be obtained a better adhesion between bracket and enamel.

Composite resins are most frequently used to bond brackets during orthodontic treatment. It is necessary to use phosphoric acid for etching, and to obtain the micropores on the enamel for mechanical bonding with the cement adhesive to obtain a satisfactory bond between the enamel and the tooth [19]. These cements have micro-mechanical mechanisms for adhesion [3], but they have enamel demineralization as a side effect. For this reason, fluoride-releasing cements were developed to inhibit the loss of minerals.

The glass ionomers can release fluoride and prevent the demineralization of the enamel, but they have lower bond strength. RMGIC were developed to improve the adhesion between brackets and the teeth and presents the advantage of a higher SBS than composite resins and fluoride-releasing due to the presence of the glass ionomers. A polyacrilic acid of 10% is used with RMGIC, because phosphoric acid is too aggressive and demineralization will be deeper. After mixing powder with liquid, the curing-mechanism is chemical, with an acid-base reaction. When light-curing begins, polymerization is initiated [28]. Previous research demonstrated that the bond strength of RMGIC is higher after 24 h, but not immediately. One of the advantages of using RMGIC for bonding brackets is that the surface of enamel is almost intact after debonding [2].

Considering all materials involved in the orthodontic devices treatment in current research, resulting in three possible ways to promote erosion [29,30]:

- the first is within the bonding structure, in our case CR and RMGIC adhesive;
- the second is on the enamel-adhesive interface;
- and the third is on the bracket-adhesive interface.

SEM microscopy evidences that the bracket-adhesive interface is very cohesive due to the bracket base equipped with adhesion pillars. The adhesive after debonding is found predominantly on the bracket base with a surface coverage between 70–85%. On the opposite, the enamel-adhesive interface after debonding has less adhesive after debonding with coverage ranging from 15 to 30%. Our SEM evidence shows that the adhesive layer presents some internal fractures during debonding, especially for CR samples exposed to Coca-Cola^TM^ and Red Bull^TM^. The RMGIC adhesive layer proves to be more cohesive without significant internal fracture after debonding. This observation shows that RMGIC is more resistant to acid erosion attempts than CR, a fact in good agreement with the data in the literature [31,32].

Debonding the orthodontic brackets from the enamel of premolars causes damage to their surfaces. The force used to mechanically debond the metallic bracket must be great enough to exceed the bond strength of the adhesive compound. Consequently, the bonding interface is sheared from the enamel, leading to the violent extraction of structural components from the enamel surface, as well as areas where the adhesive remains attached. These aspects must be investigated by a detailed microstructural investigation.

Tooth enamel is a complex and hierarchic structure based on the hydroxyapatite (HAP) nanoparticles stuck together with a protein binder. HAP nanoparticles are organized in nanostructural units with rounded shapes and diameters of 40 nm which forms the enamel nanostructure. The nanostructural units are further bonded together into the HAP prisms having a diameter of about 5 µm which represents the fine microstructure of the enamel.

SEM evidence agrees that the results of SBS testing and proves that the enamel-adhesive interface is the most affected by the shearing failure. CR adhesive presents evidence of internal fracturing under shearing forces. Both aspects are influenced by acid erosion. RMGIC prove to be more resistant than the others samples. The best situation observed by SEM and SBS is resulted from control samples stored in artificial saliva. The best results after acid environment exposure is obtained for RMGC exposed to Red Bull and the worst results were obtained for CR samples exposed to Coca-Cola. These aspects require a more detailed microscopic and topographic investigation with the AFM.

It is expected that cola is more erosive against RMGIC bonding layer than Red Bull because of the pH difference between them. However, data in the literature shows that the polymer coating over mineral particles assures good insulation against acid erosion [33]. SEM images prove that our RMGIC sample has very good insulation of the mineral filler particle. The acid liquid has to penetrate the insulation within the bonding layer to cause the weakening of the shear bond strength. Therefore, besides the pH value, the liquid viscosity is very important because it directly influences the liquid absorption within RMGIC bulk. Red Bull^TM^ is more viscous than the artificial saliva and Coca-Cola^TM^ due to a large number of dissolved vitamins [34,35], respectively it has a lower penetration potential and in consequence, it is an explanation why it assures the best SBS value of the RMGIC sample. The fact requires more investigation on the sample liquid absorption and solubility which is the subject of a further article.

However, AFM studies in the literature have shown that citric acid is more erosive than phosphoric acid [1,11]. Data in the literature also shows that Red Bull^TM^ is also hazardous for aquatic life forms [36]. This is how it can be explained why SBS was higher for the brackets bonded with RMGIC and immersed in Red Bull^TM^, which contains citric acid, and determine adverse effects in the structure of the teeth but the adhesion interface and enamel area behind it is protected by the good insulation of mineral filler [37].Coca-Cola contains phosphoric acid that can produce enamel surface modification, which appeared as irregularities in enamel morphology and will determine the decrease in SBS with a negative effect on bracket retention and on the success of orthodontic treatment. The results of SBS determinations show significant differences and demonstrate that the two acids had produced a very deep demineralization. Considering our AFM topographic and morphologic observation, we can conclude that the erosive effect of Cola exposure is in accordance with data in the literature [27] and is caused by phosphoric acid etching. The Red Bull^TM^ energy drink contains a multitude of potentially erosive ingredients, which translates into a greater aggressive upon exposure to sample surfaces when compared to Cola-exposure. The more advanced enamel demineralization, in this case, is in accordance with data in the literature [18].

Bond failure may occur within the bracket, at the bracket-adhesive interface, within the adhesive, or in the tooth-adhesive interface [27]. The presence of micro-leakage at the enamel-adhesive interface determines the appearance of white spot lesions, while micro-leakage at the adhesive-bracket interface is responsible for bracket failure [1]. Bracket failure should occur at the enamel-adhesive interface, which will make adhesive removal and polishing much easier. Bracket debonding and mechanical adhesive removal can determine iatrogenic effects including rough surface and enamel cracking or fracture. The low residual adhesive on the surface of the enamel reduces the time for polishing, is less harmful to the structural integrity of the tooth. AFM images show that the debonded samples stored in the artificial saliva preserve the best enamel fine microstructure and nanostructure. Exposure to Coca-Cola and Red Bull induces acid erosion by enlarging the unevenness that may occur on the enamel surface forming erosion depressions and alteration that generated advanced decaying with possibly weight loss. The most erosive agent for CR was Coca-Cola^TM^ and for RMGIC was Red Bull^TM^ as observed in the roughness variations presented in Figure 4 and Figure 6.

The enamel was polished after the brackets debonded, aiming for the removal of the remained adhesive and to obtain a smoother and less eroded surface. Data in the literature shows that a rough surface will determine the retention of bio-film and decrease light reflection [38]. RMGIC removal can be accomplished easily, as compared to composite resin [18]. AFM results show that the lowest roughness in each group was obtained after the polishing treatment.

The polished surface exposure to the acid environment is affected by this fact is sustained by the lower values of the roughness of these samples compared to the unpolished ones exposed to the acid environment. Even after the polishing, the AFM results shows that Coca-Cola^TM^ is more erosive for CR samples and Red Bull is most erosive for RMGIC, fact in good agreement with the data in the literature [11,14,28]. Overall, we can conclude that polishing the enamel causes a delay in acid etching compared to the unpolished samples. The correlation of all obtained results rejects both aspects of the null hypothesis.

## 4. Materials and Methods

### 4.1. Samples Preparation

Sixty premolars extracted for orthodontic reasons were used for this study. They had no caries, no surface cracks, or chemical treatment before the extraction. The teeth were cleaned with a low-speed rotary instrument and a prophylactic brush and a fluoride-free paste (Cleanic, Kerr, Kloten, Switzerland).

Roth brackets of 0.022-inch stainless steel were used. They were bonded in the third middle of the buccal surface of each tooth with the long axis parallel to the axis of the tooth. The specimens were randomly divided into two groups. Brackets in each of these groups were bonded according to the standard procedures required by the adhesives manufacturer detailed below:

Group 1: Normal metallic brackets were bonded with Transbond Plus Color Change (3M Unitek, St. Paul, MN, USA) on the vestibular surface of thirty teeth:

(1) 37% phosphoric acid was applied for 30 s, the acid was rinsed off and the enamel was dried.
(2) The primer with the adhesive was applied with a sponge for 20 s, dry easily and light-cured for 20 s.
(3) The cement resin was applied on the base of the bracket and the bracket was placed and pressed on the vestibular surface of the teeth. Removing the excess around the bracket could be difficult due to the fact that the color of tooth enamel and the cement are alike. That is why a colored orthodontic cements system has been created, which changes the color during polymerization as observed by Turkkahraman et al., 2010 [13]. After the excess was removed, it was light cured for 20 s, with 5 s light incidence over each marginal side of it.

Group 2: The brackets were bonded with Fuji Ortho LC (GC Company, Tokyo, Japan) capsules on the surface of thirty teeth. Bracket bonding takes about 1–2 min (depending on the environment temperature) after the RMGIC was mixed, or the adhesive will be rough [14].

(1) Thirty teeth were etched using a 10% acid polyacrilic enamel conditioner. The conditioner was applied for 20 s and the teeth were rinsed and dried. We did not use phosphoric acid with RMGIC, because this type of acid will demineralize too profoundly.
(2) The capsules containing the RMGIC (Fuji Ortho LC) were activated and triturated at 4000 rpm for 8 s. Capsules were placed in the GC Capsule Applier to place the adhesive on the base of the bracket
(3) RMGIC was applied and the bracket was placed on buccal enamel and pressed firmly into place. The excess of adhesive material may increase the retention of dental plaque and will determine the increase in the incidence of gingivitis and caries according to Naranjo et al., 2006 [15]. The excess was removed with a sharp scaler and the bracket was light-cured for 20 s.

The acid exposure protocol was identical for all tested samples as follows. All samples were kept in artificial saliva at 37 Celsius for 24 h to allow the complete polymerization. We used their own prepared artificial saliva produced at the Department of Polymer Composites, Institute of Chemistry “Raluca Ripan”, Babes-Bolyai University. It contains Na_2_HPO_4_, NaHCO_3_, CaCl_2_, and HCl in an aqueous solution with pH = 7. Each group was divided into three other groups, with ten teeth in every group.

Group A: was kept in artificial saliva, pH = 7.

Group B: was immersed in Coca-Cola^TM^ (pH = 2.5) for 15 min, three times a day, with equal intervals. This procedure was repeated for 20 days.

Group C: the teeth were immersed in Red Bull^TM^ (pH = 3.3), three times a day for 15 min over 20 days. Teeth were kept in artificial saliva for the rest of the time. Artificial saliva was refreshed daily. After 20 days, all teeth were mounted vertically in acrylic blocks up to the clinical crown level, so the force could be applied to the bracket-tooth interface parallel to the buccal tooth surface in an occlusion apical direction. Duracryl Plus, from Spofa Dental (Kerr, Kloten, Switzerland), was used.

### 4.2. Shear Bond Strength Test

The shear bond strength (SBS) test was effectuated with the ASTM D638 test method according to the data in the literature [16,17] using the Lloyd LR5k Plus dual-column mechanical testing machine (Ametek/Lloyd Instruments, Germany), force capacity 5 kN. The universal materials testing machine features an electronic system to test and measure compression. The used load used was 0.5 N with a crosshead speed of 1 mm/min in the occluso-gingival direction. The data collected were processed using NEXYGEN Plus 3.0 software.

The value of SBS for each group is the mean of 10 mechanical tests (n = 10). Further ANOVA and Tukey testing was run on the datasets for post hoc comparison between the groups, and the significance level was set at α = 0.05, using the Origin2019b Graphing and Analysis software, Microcal Co., Northampton, MA, USA.

### 4.3. Scanning Electron Microscopy SEM

Tooth enamel was studied using scanning electron microscopy (SEM Inspect™ S, FEI, Hillsboro, OR, USA) after the shear bond test, bracket debonding, and polishing, the surface morphology of the. This electron microscope generates the image of a sample surface scanning with a focused electron beam. The electrons interact with the enamel atoms and thus produce various signals that provide information regarding the surface topography and chemical composition of the tooth. The specimens were removed from the artificial saliva they were stored in, patted dry with filter paper and analyzed in a low vacuum, 4.5 spot and 25.00 kV, 100× magnifications.

### 4.4. Atomic Force Microscopy AFM

This study analyzes the tooth enamel surface at the precise place where orthodontic brackets had been previously attached. The samples are divided into two groups: “CR-Enamel” and “RMGIC-Enamel”. The specimens were sectioned parallel to the enamel surface to ensure optimal positioning for the AFM investigation. We prepared several sets of specimens to accommodate the required testing. As such, the enamel samples include debonded surface sections, prior to polishing, and uniform surface sections, after polishing.

A specimen of both before and after polishing samples were kept as control samples, while the rest were divided into two groups that were exposed to an acid etching medium: one group was exposed to a Coca-Cola^TM^ beverage (acidifier-phosphoric acid) and another group was exposed to the Red Bull^TM^ energy drink (acidifier-citric acid) and a vitamin mixture. The acid exposure for each group follows the protocol described in Section 4.1.

All samples were then cleansed in bi-distilled water and stored in the artificial saliva in individual containers at room temperature. Each sample was extracted from their container, cleansed with bi-distilled water, dried with filter paper, and mounted onto the required AFM support. After the AFM investigation, each sample was reinserted into itsartificial saliva container.

We aim to observe the evolution of the morphology and topography of the sample surfaces in response to the applied treatments. During the first stage, we evaluate if there are any improvements in the surface quality of the enamel samples after polishing. During the second stage, we evaluate the effect of acid etching on the samples, before and after any polishing is applied.

The null hypothesis of this study has two conjectures: the first is that the effect of acid etching does not depend on the polishing treatment, and the second is that there is no difference between the degree of degradation caused by Coca-Cola^TM^ and Red Bull^TM^.

The atomic force microscopy (AFM) analysis was effectuated using a JEOL microscope (JEOL JSPM 4210, JEOL, Tokyo, Japan). All samples were evaluated in tapping mode using NSC 15 cantilevers produced by MikroMasch, Bulgaria Headquarters, Sofia. The cantilever resonant frequency was 330 kHz, and the spring constant was 48 N/m. In accordance with data in the literature [20,39,40,41,42], the topographic images scanned and recorded were as follows: a 5 µm × 5 µm area for the fine microstructure of enamel and a 1 µm × 1 µm area for the enamel nanostructure. Three separate macroscopic areas were scanned for each sample. The images were processed using the WinSPM 2.0 JEOL software (WinSPM 2.0 JEOL, Tokyo, Japan) in accordance with standard procedures, presenting 2D topographic images, 3D images and the Ra and Rq surface roughness parameters were measured. *Ra* represents the arithmetic average of the profile height and is described by Equation (1) and *Rq* root mean square of the profile height and is described by Equation (2):

(1) $$Ra=\frac{1}{l_{r}}\int_{0}^{l_{r}}\left|z\left(x\right)\right|dx,$$

and,

(2) $$Rq=\sqrt{\frac{1}{l_{r}}\int_{0}^{l_{r}}\left|z{\left(x\right)}^{2}\right|dx}.$$

where: *l* is the profile length and *z* is the height at *x* point. Both *Ra* and *Rq* are important for various research applications.

The 3D images are also called 3D profiles; they are a graphic representation of the depth profile of the enamel surface, closely related to the measured values of surface roughness.

For each test group, the Ra and Rq values represent the mean for three measurement areas (n = 3). The data were then run through the ANOVA One-Way test to compare the effect of acid etching both before and after polishing, and the significance level was set at α = 0.05, using the Origin 2019b Graphing and Analysis software, Microcal Co., Northampton, MA, USA.

## 5. Conclusions

The acid environment influences the shear bond strength of the adhesion layer of CR and RMGIC samples because of the pH values associated with the micro-structural aspects (e.g., pores and fissures) allowing the erosive liquid penetration into the bonding material. The proper polymeric insulation of the mineral filler particle assures the proper resistance to the erosive agent penetration on the inside of the bonding layer and preserves the bracket retention. RMGIC samples prove to be more resistant in certain conditions than CR. The enamel surface after CR debonding is more affected by Coca-Cola^TM^ due to the phosphoric acid content and the enamel after RMGIC debonding was more affected by Red Bull^TM^ due to the citric acid content. Polishing treatment of the debonded enamel areas further assures a good resistance against the acid erosive effect of both Coca-Cola^TM^ and Red Bull^TM^.

## Figures and Tables

**Table 1:** Shear bond test results.

Mechanical Properties	Saliva		Coca-Cola^TM^		Red Bull^TM^	
	Mean	SD	Mean	SD	Mean	SD
CR						
Bond Strength, MPa	5.20774	1.96879	2.72076	0.77658	2.90572	1.49381
Maximum Load, N	67.5786	23.91672	32.96726	22.66505	35.92676	18.51022
Breaking Load, N	60.34059	31.61737	30.044	18.14057	31.888897	15.86262
RMGIC						
Bond Strength, MPa	7.65976	2.3793	3.53586	1.10649	3.62436	2.38791
Maximum Load, N	86.92681	24.5682	41.42943	14.38154	53.40967	36.83688
Breaking Load, N	82.48911	26.11797	31.19834	10.23796	50.90918	38.59967

**Table 2:** Surface roughness statistical analysis for the samples after debonding.

	CR + Saliva	CR + Cola	CR + Red Bull	RMGIC + Saliva	RMGIC + Cola	RMGIC + Red Bull
**Enamel fine microstructure**						
Ra, nm	66.90	117.66	115.56	86.90	116.83	133.30
P1		0.08	0.08		0.46	0.46
Rq, nm	87.63	151.66	150	105.50	145.66	170.30
P2		0.06	0.06		0.36	0.36
**Enamel nanostructure**						
Ra, nm	39.43	46.6	55.7	25.56	35.26	51.26
P1		0.24	0.24		0.21	0.21
Rg, nm	48.63	57.83	69	36	42.53	63.83
P2		0.24	0.24		0.25	0.25

**Table 3:** Surface roughness statistical analysis for the samples after polishing.

	CR + Saliva	CR + Cola	CR + Red Bull	RMGIC + Saliva	RMGIC + Cola	RMGIC + Red Bull
**Enamel fine microstructure**						
Ra, nm	66	90.03	105.13	69.53	75.2	133.43
P1		0.40	0.40		0.24	0.24
Rq, nm	77.56	117.63	131.33	86.43	94.7	164.66
P2		0.41	0.41		0.20	0.20
**Enamel nanostructure**						
Ra, nm	10.4	15.16	16.70	14.26	18.23	21.23
P1		0.22	0.22		0.29	0.29
Rg, nm	13.28	19.10	21.23	18.03	23.10	26.26
P2		0.22	0.22		0.28	0.28

## Data Availability

The data presented in this study are available on request from the corresponding author.
